# TIMP-1 as well as Microvessel Invasion and High Nuclear Grade Is a Significant Determinant Factor for Extension of Tumor Diameter in Localized RCC

**DOI:** 10.1155/2016/5035127

**Published:** 2016-02-25

**Authors:** Nozomu Kawata, Kenya Yamaguchi, Tomohiro Igarashi, Satoru Takahashi

**Affiliations:** Department of Urology, Nihon University School of Medicine, 1-6 Kanda-Surugadai, Chiyoda-ku, Tokyo, Japan

## Abstract

*Objectives*. To clarify what kind of pathological factor is necessary for the extension of tumor diameter in localized RCC, we studied localized RCC patients.* Methods*. We retrospectively reviewed medical records of 237 RCC patients in our institute who underwent nephrectomy. We performed immune histological analysis of MMP-2, MMP-9, TIMP-1, TIMP-2, and MT-MMP-1 for all samples.* Results*. Among the clinicopathological factors, multivariate analysis revealed nuclear grade; TIMP-2 and MT-MMP-1 were independent prognostic factors of localized RCC (risk ratio 1.50, *p* = 0.037, risk ratio 1.12, *p* = 0.008, and risk ratio 1.84, *p* = 0.045, resp.). By the multiple logistic regression analysis among pT1a versus pT1b, TIMP-1 was an independent factor (risk ratio 3.30, *p* = 0.010) whereas all pT1 versus pT2a and all pT1 + pT2a versus pT2b high nuclear grade (risk ratio 5.15, *p* = 0.0015) and Micro vessel invasion (MVI, risk ratio 3.08, *p* = 0.002) were independent factors. For all pT1 + pT2a versus pT2b, nuclear grade (risk ratio 3.39, *p* = 0.020) and MVI (risk ratio 2.91, *p* = 0.018) were independent factors.* Conclusion*. Higher expression of TIMP-1 is necessary for advancement tumor diameter from pT1a to pT1b, and a process of tumor diameter extension beyond pT1 and pT2a category needs presence of MVI and high nuclear grade.

## 1. Introduction

Recently, Frank et al. [1] and Klatte et al. [2] proposed a subclassification of T2 RCC into pT2a and pT2b according to tumor diameter with a cutoff of 10 cm.

Based on their reports, the 7th edition of TNM classification [3], threshold value between T1 and T2 RCC was divided into T2a (up to 10 cm) and T2b (more than 10 cm) [3]. Lee et al. [4] reported that local control may be achieved in surgical management of contemporary patients with RCC of 4 cm or less either by radical or nephron sparing surgery, and, in addition, local recurrence rate after nephron sparing surgery was 0–12%. The rate decreases to 0% to 3% for microscopically organ confined disease and 0% to 5% for small renal tumors [5]. It is well known that renal cell carcinoma with a diameter of more than 10 cm has high potential to cause distant metastasis and generally recommended surgical procedure is radical nephrectomy [1].

Previously, we reported that systemic symptoms of RCC have a strong significant relationship with the expression of matrix metalloproteinase 9 (MMP-9) [6]. It is well known that both MMPs (matrix metalloproteinases) and TIMPs (tissue inhibitors of metalloproteinases) play an important role in the progression of RCC. However, there are no reports examining the relationships among tumor diameter and MMPs and TIMPs. To clarify what kind of clinicopathological feature is necessary for extension of the tumor diameter, we studied localized RCC patients.

## 2. Material and Method

Between January 1988 and December 2003, a total of 237 patients had underwent radical nephrectomy for localized renal cell carcinoma at Nihon University Itabashi or Surugadai Hospital. Patients consisted of 176 males and 61 females, mean age of 60 (33–83) and 58 (25–82), respectively. The average postoperative follow-up period was 61 ± 3.6 months. All patients underwent preoperative chest and abdominal contrast enhanced CT, and bone scan if required. Pathological stages were determined according to the TNM classification of malignant tumors [3].

Tumors were classified as pT1a, pT1b, pT2a, and pT2b in 94 (40%), 74 (31%), 43(18%), and 26 (11%) cases, respectively.

The nuclear grade of RCC was determined using the criteria proposed by Fuhrman et al. [7]. Since several studies found no significant difference in survival results between patients with Grade 1 versus 2 tumors and those with Grade 3 versus 4 tumors [8], a total of 237 patients were divided into two groups according to nuclear grade: a low nuclear grade group (Grades 1 and 2, 190 patients) and a high nuclear grade group (Grades 3 and 4, 47 patients).

Microvessel invasion (MVI) was defined as a tumor infiltration locally through the intact vessel wall including the endothelium, leading to free extension of cancer cells into the lumen [9].

The maximum tumor diameter (MTD) was confirmed by pathological specimens. We applied immunohistochemistry on the cut surface of tumor with no necrosis nor intratumoral hemorrhage.

**Figure 1:**
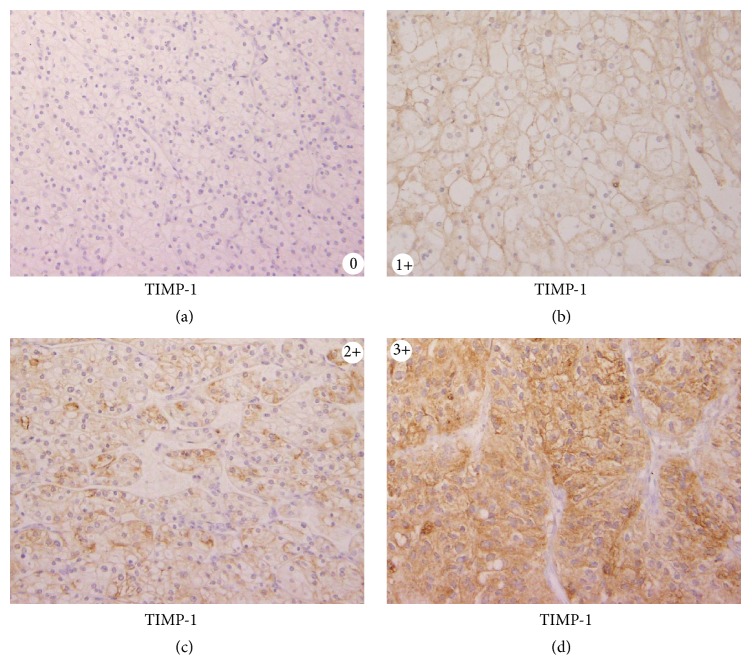
0 indicates the absence of immune staining or faint membranous staining of rare tumor cells; 1+ indicates membranous staining in most tumor cells; 2+ indicates diffuse membranous and/or cytoplasmic staining in groups of tumor cells; and 3+ indicates significant cytoplasmic staining in most tumor cells. For the evaluation of immune histochemical staining, intensities of 2+ and 3+ were considered strong expressions of each protein.

The immunohistochemical study for MMP-2, MMP-9, TIMP-1, TIMP-2, and MT-MMP-1 was performed by methods we previously reported [6]. For evaluation of immunohistochemical staining, staining intensities of 2+ and 3+ were considered strong expressions of each protein (Figure 1) [6].

Cancer-specific survival (CSS) was defined as the interval from initial surgery to death and was calculated by the method of Kaplan and Meier. Statistical significance was determined by the log-rank test. Cox multivariate analysis was performed to determine any independent predictive values.

To determine the relationships between T categories and 8 pathological features of RCC (histopathological type, nuclear grade, MVI, MMP-2, MMP-9, TIMP-1, TIMP-2, and MT-MMP-1), we compared the quantitative results using a multiple logistic regression analysis. Intergroup differences were considered statistically significant at *p* < 0.05. All analyses were performed using JMP4.0 (SAS Institute, Cory, NC, USA).

The study using these specimens was performed under the approval of Nihon University School of Medicine Ethics Board (IRB number 106-1).

## 3. Results

Among the tumors, 194 (82%) were conventional clear cell carcinomas, 42 (17%) were papillary carcinomas, and 1 (0.3%) was a chromophobe carcinoma. Tumors were classified as pT1a, pT1b, pT2a, and pT2b in 94 (40%), 74 (31%), 43 (18%), and 26 (11%) cases, respectively. The median tumor diameter was 50 mm (15–250 mm). Among a total of 237 patients, 190 were classified as having a low nuclear grade (Grades 1 and 2), whereas 47 as having a high nuclear grade (Grades 3 and 4).

**Figure 2:**
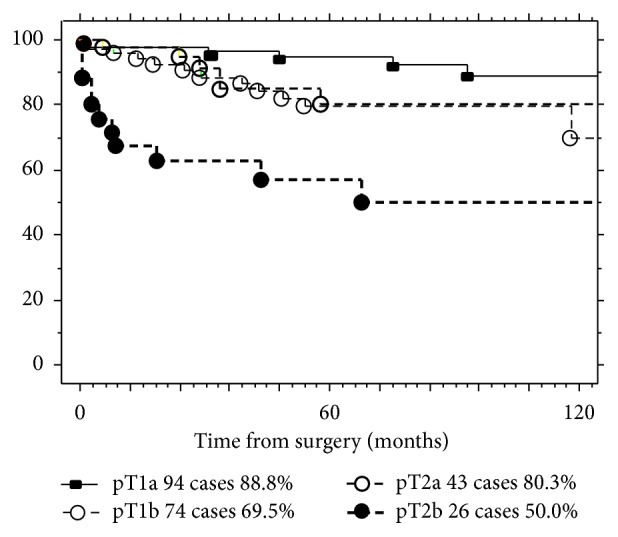
Cancer-specific survival rate according to the pT category.

The cancer-specific 10-year survival rates were 88.8%, 69.5%, 80.3%, and 50.0% for pT1a, pT1b, pT2a, and pT2b, respectively (Figure 2,  *p* < 0.001).

With respect to the cancer-specific mortality, the univariate analysis showed no significance for pT1a versus pT2a and TIMP-1 and TIMP-2 as a determinant factor, while the remaining 8 factors were significant factors of postoperative specific mortality of 247 patients (Table 1). By the multivariate analysis of clinicopathological factors, nuclear grade, TIMP-2, and MT-MMP-1 were independent prognostic factors (risk ratio 1.50, *p* = 0.037, risk ratio 1.12, *p* = 0.02, and risk ratio 1.84, *p* = 0.045, resp.) (Table 1).

We compared the clinicopathological factors in three categories: pT1a versus pT1b, all pT1 versus pT2a, and all pT1 + pT2a versus pT2b. By the Cox multivariate analysis (Table 2), among the pT1a versus pT1b group, TIMP-1 was an independent factor (risk ratio 3.30, *p* = 0.010). For pT1 versus pT2a, both nuclear grade (risk ratio 5.15, *p* = 0.0015) and MVI (risk ratio 3.08, *p* = 0.002) were independent factors. For the remaining pT1 + pT2a versus pT2b, both nuclear grade (risk ratio 3.39, *p* = 0.020) and MVI (risk ratio 2.91, *p* = 0.018) were significant factors.

## 4. Discussion

Once MMPs are stimulated, they are susceptible to prohibition by the general serum proteinase inhibitor *α*2-macroglobulin and by a family of specific tissue inhibitors (TIMPs). On the other hand, TIMPs have been frequently reported that they may be multifunctional, because of additional effects on cell growth and apoptosis. These activities appear to be distinct from their MMP inhibitory capabilities in some cases [10].

Previously, we reported that high expression levels of MMP-9 were associated with poor prognosis of RCC [11]. Basically, TIMPs are known to inhibit MMP activity by forming a complex with active MMPs and are believed to be specific for enzymes of this family, such as TIMP-1 with MMP-9 and TIMP-2 with MMP-2 [12]. Members of the TIMP family have also been associated with cancer. In several cases, malignant tumors have elevated TIMP levels rather than decreased levels [12].

MMP-9 has a significant relationship with high nuclear grade RCC and was found to be an independent prognosticator by multivariate analysis. Furthermore, nuclear grade and TIMP-2 were independent prognostic factors among the incidental RCC patients [13].

With regard to MVI, Ishimura reported that MVI is not a significant prognostic factor in localized RCC patients; on the other hand, it is the only significant prognostic factor of disease free recurrence after radical operation for patients with pT1 and pT2 disease [14]. Additionally, Dall'Oglio et al. showed a significant relationship between MVI and clinical stage. In 95 tumors below 4 cm in diameter, MVI was detected in 11 (12%), while in 74 tumors of 4.1–7 cm, MVI was detected in 20 (27%), and in 61 of over 7 cm, 28 (48%) had MVI [15].

Previously we reported that cancer-specific 5-year survival was 45.0% for patients with high nuclear grade tumor (Grade 3.4) and 83.3% for patients with low nuclear grade tumor (Grade 1.2) (*p* < 0.001) [16]. Zhang et al. reported a significant correlation between tumor size and nuclear grade. By their report, tumor diameters of G1, G2, and G3 tumors were significantly different (3.27 ± 1.46 cm, 4.87 ± 2.23 cm, and 7.39 ± 3.11 cm, *p* < 0.05). Tumors with larger diameter were prone to have higher nuclear grade. These results were consistent with ours [17].

## 5. Conclusion

In conclusion, higher expression of TIMP-1 is necessary for advancement of tumor diameter from pT1a to pT1b, and a process of tumor diameter extension beyond pT1 category needs the presence of MVI and high nuclear grade.

## Figures and Tables

**Table 1:** Predictors of localized 237 RCC cases postoperative specific mortality.

Categories	Univariate analysis		Multivariate analysis	
	Hazard ratio (95% CI)	*p* value	Hazard ratio (95% CI)	*p* value
pT1a versus pT1b 94 versus 74	2.38 (1.02–5.55)	0.046	1.35 (0.96–1.88)	0.086
pT1a versus pT2a 94 versus 43	1.40 (0.49–4.0)	0.53	1.07 (0.93–1.42)	0.74
pT1a versus pT2b 94 versus 26	4.0 (1.51–1.020)	0.005	1.09 (0.66–1.85)	0.71
Clear versus nonclear 190 versus 47	2.05 (1.00–4.019)	0.048	1.12 (0.77–1.65)	0.53
Nuclear Grades 1 and 2 versus 3 and 4 190 versus 47	1.79 (1.76–6.84)	<0.001	1.50 (1.03–2.22)	0.037
MVI (−) versus (+) 154 versus 83	1.95 (0.92–2.94)	0.025	1.04 (0.33–1.21)	0.94
MMP-2 weak versus strong 82 versus 155	3.69 (1.43–9.52)	0.0069	1.26 (0.30–5.23)	0.74
MMP-9 weak versus strong 181 versus 56	4.29 (2.17–8.16)	<0.0001	2.88 (0.92–2.94)	0.75
TIMP-1 weak versus strong 42 versus 195	2.52 (0.77–8.26)	0.12	1.014 (0.709–1.45)	0.34

TIMP-2 weak versus strong 201 versus 36	2.07 (0.99–4.31)	0.052	1.12 (1.36–3.29)	**0.020**
MT-MMP-1 weak versus strong 194 versus 43	3.44 (1.73–6.84)	0.005	1.84 (1.21–2.82)	**0.045**

**Table 2:** Correlation between pT category and pathological features with localized 237 RCC cases.

	pT1a versus pT1b	pT1a and pT1b versus pT2a	pT1a, pT1b, and pT2a versus pT2b
	Odds ratio (95% CI) *p* value	Odds ratio (95% CI) *p* value	Odds ratio (95% CI) *p* value
	94 versus 74	168 versus 43	211 versus 26
	Cases of each category	Cases of each category	Cases of each category
Cell type	2.43 (0.85–6.89) 0.09	2.28 (0.76–6.82) 0.13	2.32 (0.84–6.36) 0.10
Clear versus others	139 versus 29	175 versus 36	190 versus 47
Nuclear grade	1.79 (0.57–5.56) 0.31	**5.15 (1.87–14.2) 0.0015**	**3.39 (1.20–9.58) 0.020**
Low versus high	147 versus 21	**176 versus 35**	**190 versus 47**
MVI	1.95 (0.92–4.16) 0.08	**3.08 (1.47–6.48) 0.002**	**2.91 (1.19–7.11) 0.018**
(−) versus (+)	123 versus 45	**144 versus 67**	**154 versus 83**
MMP-2	1.14 (0.55–2.36) 0.71	2.13 (0.90–5.0) 0.84	1.24 (0.41–3.70) 0.70
Weak versus strong	60 versus 108	76 versus 135	82 versus 156
MMP-9	1.79 (0.65–4.90) 0.205	1.47 (0.55–3.94) 0.43	1.31 (0.40–4.21) 0.64
Weak versus strong	134 versus 34	163 versus 48	181 versus 56
**TIMP-1**	**3.30 (1.32–8.26) 0.010**	1.18 (0.43–3.26) 0.74	1.59 (0.49–5.10) 0.42
Weak versus strong	**31 versus 137**	37 versus 174	42 versus 195
TIMP-2	1.41 (0.48–4.13) 0.53	2.14 (0.65–6.99) 0.84	3.03 (0.71–12.98) 0.13
Weak versus strong	142 versus 26	178 versus 33	201 versus 36
MT-MMP-1	1.93 (0.65–5.71) 0.096	2.28 (0.76–3.50) 0.15	1.98 (0.60–6.54) 0.26
Weak versus strong	144 versus 26	176 versus 35	194 versus 43

MVI: microvascular invasion.
